# Celebrating 50 years of translational neuroscience in Kuopio

**DOI:** 10.1002/nep3.70067

**Published:** 2026-10-26

**Authors:** Jukka Jolkkonen

**Affiliations:** ^1^ A.I. Virtanen Institute for Molecular Sciences University of Eastern Finland Kuopio Finland

The first professorship in neurology at the University of Kuopio, Finland, was established in 1976, marking the beginning of what has since developed into a strong neuroscience research environment. This 50‐year journey was celebrated in Kuopio on June 15–17, 2026, bringing together more than 300 researchers and clinicians to reflect on past achievements and discuss emerging directions in neuroscience.

From the very beginning, research in Kuopio has focused particularly on epilepsy, cerebrovascular diseases, and memory disorders. Research on cerebrovascular and memory disorders benefited from the legacy of the North Karelia Project, which demonstrated that population‐level lifestyle changes could substantially reduce cardiovascular risk factors and, consequently, cardiovascular mortality in Eastern Finland.

Importantly, neuroscience research in Kuopio has had a strong translational orientation. Although the term *translational research* was not yet widely used, the underlying concept was already well established: experimental research was closely linked to clinically relevant questions and patient care. International training played an important role in this development. Young researchers were encouraged to work abroad to acquire new methodological expertise and scientific knowledge, while these visits also laid the foundation for many long‐lasting international collaborations.

Supported by strong scientific leadership and generations of talented and ambitious early‐career researchers, Kuopio is today an internationally recognized multidisciplinary center for neuroscience. In this review, I highlight some selected scientific hot topics from the 50th anniversary symposium that illustrate this journey and may be of particular interest to readers of *Neuroprotection*.

## 1 MICROGLIA–NEURON INTERACTION IN BRAIN DISEASES

Michael Heneka (University of Luxembourg) discussed the evolving role of microglia and innate immunity in Alzheimer's disease (AD). Increasing evidence indicates that immune activation begins early, alongside amyloid‐β (Aβ) accumulation, and persists throughout disease progression. Importantly, microglia do not remain in a single activation state but undergo changes in cell fate and function as AD progresses. A key mechanism linking inflammation and amyloid pathology is the NLR family pyrin domain containing 3 (NLRP3) inflammasome. Aβ activates the microglial NLRP3 inflammasome, triggering the release of apoptosis‐associated speck‐like protein containing a CARD (ASC) specks that promote further Aβ aggregation, thereby establishing a self‐amplifying cycle of amyloid accumulation and neuroinflammation. Heneka also highlighted tunneling nanotubes as direct intercellular transport routes between microglia and neurons.^1^ These connections may allow microglia to remove pathological protein aggregates from neurons while, in the opposite direction, delivering functional mitochondria to stressed neurons. Thus, microglia may have both detrimental inflammatory and beneficial supportive functions in AD. Overall, AD involves a dynamic and progressively changing neuroimmune response. Consequently, successful immune‐targeted therapies will require precise understanding of which immune processes should be targeted, where, and at what stage of the disease.

Soyon Hong (University College London) continued by discussing neuro–glia–immune mechanisms underlying synapse loss in neurodegenerative disease. Brain immunity is increasingly recognized as an important driver of disease risk and progression, and synapse loss is one of the strongest pathological correlates of cognitive decline in AD. Mechanisms normally involved in developmental synaptic pruning may become aberrantly reactivated in disease. In particular, microglia mediate the removal of vulnerable synapses, with the complement protein C1q marking synapses for elimination.^2^ Additional “eat‐me” signals include phosphatidylserine, which can be exposed at synapses in response to Aβ oligomers. An important question is what determines which synapses become vulnerable. Microglia dynamically respond to changes in neuronal activity, and synapse loss in AD shows a spatial and temporal relationship with neuronal hyperactivity. Increased neuronal activity can promote the C1q‐dependent elimination of neighboring, less active synapses in the adult hippocampus. Thus, synapse vulnerability is context‐dependent and determined by coordinated neuro–glia–immune interactions rather than by microglia alone. Interestingly, spatial transcriptomics implicated B cells and plasma cells in regions undergoing activity‐dependent synaptic remodeling. These cells localized to sites of neuronal activity, while IgM colocalized with C1q at vulnerable hippocampal synapses. Together, these findings suggest that neuronal hyperactivity can engage an innate–adaptive immune axis at synapses, linking neuronal activity, complement activation, microglial synapse elimination, and adaptive immune responses in AD.

## 2 UNIQUE WINDOW TO THE BRAIN

Alejandra Sierra López and Tarja Malm (University of Eastern Finland) presented a multiscale approach to understanding epileptogenicity in patients with drug‐resistant epilepsy undergoing epilepsy surgery. Surgical tissue is transferred directly from the operating room to the laboratory, enabling comprehensive characterization of the same specimen using complementary methods. One part of the resected tissue is processed for histopathology, ex vivo high‐resolution magnetic resonance imaging, and electron microscopy, followed by multilevel integration and three‐dimensional reconstruction. Fresh tissue is also used to assess circuit‐level dysfunction using multielectrode array (MEA) recordings,^3^ while single‐neuron electrophysiology combined with biocytin filling enables detailed characterization of neuronal function and morphology. These approaches are complemented by single‐nucleus sequencing, with spatial transcriptomics currently being incorporated into the workflow. As an example, analysis of focal cortical dysplasia type IIIa reveals evidence of active synaptic remodeling. Ultimately, integration of these multimodal datasets across molecular, cellular, circuit, and tissue levels aims to identify the mechanisms that render human brain tissue epileptogenic.

Evan Macosko (Broad Institute of Massachusetts Institute of Technology and Harvard) presented data from a unique cohort of 295 cortical biopsies from patients with idiopathic normal pressure hydrocephalus (iNPH), collected and characterized by the Kuopio team.^4^ iNPH is a treatable neurological disorder characterized by gait and balance disturbances and cognitive impairment. During shunt surgery, a small cortical biopsy can be obtained for diagnostic purposes. Importantly, approximately 25% of these patients show Aβ pathology and 5% tau pathology, providing a unique opportunity to investigate early AD pathology in living patients with longitudinal clinical follow‐up. The aim is to integrate molecular and cellular data from these biopsies with postmortem human and animal datasets, increasing statistical power and enabling comparative analyses across disease stages and experimental models. The cohort also provides an opportunity to investigate cognitive resilience to AD pathology. In particular, cognitive annotation of amyloid‐responsive microglial states revealed that specific microglial states show stronger associations with cognitive responses to amyloid pathology than those of any other cell type, highlighting microglia as potential determinants of cognitive resilience or vulnerability in early AD.

## 3 GENE THERAPY IN EPILEPSY: FROM BENCH TO BEDSIDE

Matthew Walker (University College London) discussed gene therapy as a potential treatment for drug‐resistant epilepsy. Epilepsy affects approximately 1% of the population, with 30%–40% of patients remaining drug‐resistant and only a small proportion being suitable for epilepsy surgery. Viral vector‐based gene therapy therefore offers an alternative strategy for patients with focal epilepsy.^5^ Using cell‐specific promoters, such as Ca^2+^/calmodulin‐dependent protein kinase II (CaMKII) to target excitatory neurons, neuronal excitability can be reduced by overexpression of the potassium channel Kv1.1. This approach has shown efficacy without apparent impairment of motor or cognitive function in several experimental models, including temporal and occipital lobe epilepsy, epilepsia partialis continua, and focal cortical dysplasia. A Phase I/IIa clinical trial is planned.

A major challenge is dose optimization, balancing insufficient transduction against excessive expression. More refined approaches include autoregulatory gene therapy, chemogenetics, and inducible promoters that restrict therapeutic activity to hyperactive neurons or periods of increased neuronal activity. Additional challenges include potential immune responses to foreign proteins and, for chemogenetic approaches, identification of suitable drug–receptor combinations.

## 4 THE CHANGING LANDSCAPE OF STROKE IN YOUNG ADULTS AND CHILDREN

Although stroke is typically considered a disease of older age, Jukka Putaala (Helsinki University Central Hospital) highlighted the increasing importance of stroke in young adults, defined here as individuals under 50 years of age.^6^ The incidence of young‐onset stroke is increasing globally, yet its underlying cause often remains unclear. Nearly half of ischemic strokes in young adults are classified as cryptogenic, emphasizing the need to identify previously unrecognized risk factors and mechanisms. The multicenter Searching for Explanations of Cryptogenic Stroke in the Young: Revealing the Etiology, Triggers, and Outcome (SECRETO) study, involving 19 European sites, was established to identify novel risk factors and causal mechanisms of cryptogenic stroke in young adults. The findings indicate that traditional vascular risk factors contribute particularly to cryptogenic stroke without patent foramen ovale (PFO), whereas non‐traditional factors appear to be more important in patients with PFO. Migraine with aura has emerged as a prominent factor, particularly in women. Behavioral and lifestyle‐related risk factors, including smoking, unhealthy diet, heavy alcohol consumption, physical inactivity, and obesity, may also contribute, together with metabolic disorders such as Type 2 diabetes. Depression has also been associated with an increased risk of young‐onset stroke. In addition, infections and chronic inflammatory conditions, including periodontitis and infections preceding stroke, have been implicated. These observations suggest that understanding stroke in young adults requires consideration of the broader human exposome and the combined effects of lifestyle, environmental, infectious, and traditional vascular risk factors.

Silva Lõo (Helsinki University Central Hospital) continued with the diagnosis and management of cerebrovascular disease in children.^7^ Although childhood stroke is rare, its incidence and prevalence are increasing, with the highest occurrence during the first 2 years of life. Stroke in children remains a serious condition, with approximately 10% mortality and a 20%–30% risk of recurrence. Risk factors are heterogeneous and often overlapping, including maternal, fetal and neonatal factors as well as other acquired conditions. Major advances in the field have come from an improved understanding of disease mechanisms, recovery and brain plasticity, together with developments in neurovascular imaging. International collaboration through the International Pediatric Stroke Study (IPSS) network, comprising more than 100 sites, has substantially strengthened research in this relatively rare condition. The Childhood Arterial Stroke Classification and Diagnostic Evaluation (CASCADE) classification has further provided a standardized approach to the diagnosis and classification of childhood arterial ischemic stroke. Outcome depends strongly on the location and extent of brain injury. Nevertheless, many children recover well, reflecting both recovery of pre‐existing functions and the development of compensatory functions. Childhood stroke requires early identification of at‐risk children, advanced neurovascular imaging, timely reperfusion therapies, and optimized rehabilitation, supported by close multidisciplinary collaboration across pediatric and adult stroke services.

## 5 DEMENTIA PREVENTION: INNOVATIVE MULTIMODAL INTERVENTIONS

Miia Kivipelto (Karolinska Institutet/University of Eastern Finland) highlighted the growing evidence for dementia risk reduction, building on early work demonstrating the importance of midlife vascular risk factors for AD.^8^ Current estimates suggest that approximately 45% of dementia cases may be attributable to potentially modifiable risk factors. The spectrum of recognized risk factors has also expanded to include, for example, hearing loss and its associated social isolation, with hearing rehabilitation showing potential to reduce cognitive decline in at‐risk individuals.

A major focus is precision prevention through multidomain interventions targeting several risk factors and biological mechanisms simultaneously. The Finnish Geriatric Intervention Study to Prevent Cognitive Impairment and Disability (FINGER) trial provides a key example, demonstrating that a multidomain lifestyle intervention can reduce cognitive decline and provide broader health benefits, including a reduced risk of chronic diseases. This approach has expanded into the World‐Wide FINGERS network, now involving 73 countries and approximately 10,000 participants across multiple trials. Prospective harmonization, data sharing, and large‐scale analyses, including AI‐FINGERS, aim to further develop individualized prevention strategies.

The field is now moving toward next‐generation, tailored multidomain interventions, including combinations of lifestyle modification with pharmacological approaches, such as FINGER plus metformin. Overall, multidomain interventions offer a feasible strategy for preventing cognitive decline across the lifespan, while future progress will depend on precision prevention, innovative combination trials, and effective implementation at both individual and population levels.

## 6 FUTURE PERSPECTIVES

The past 50 years have witnessed remarkable advances in neuroscience, and Kuopio has been an active part of this development. Building on a strong tradition of translational and multidisciplinary research, the coming decades offer new opportunities to deepen our understanding of brain diseases and, importantly, to translate scientific discoveries into effective treatments. The next 50 years will hopefully bring much‐needed new and more effective treatments for neurological disorders.

## AUTHOR CONTRIBUTIONS

**Jukka Jolkkonen:** Writing—original draft; Writing—review and editing.

## CONFLICT OF INTEREST STATEMENT

Jukka Jolkkonen is a member of the journal's Editorial Board. The contents of this manuscript represent his personal views. He was not involved in the peer‐review process or any editorial decisions concerning this manuscript.
